# AgRP neuron hyperactivity drives hyperglycemia in a mouse model of type 2 diabetes

**DOI:** 10.1172/JCI189842

**Published:** 2025-05-15

**Authors:** Yang Gou, Micaela Glat, Vincent Damian, Caeley L. Bryan, Bao Anh Phan, Chelsea L. Faber, Arikta Trivedi, Matthew K. Hwang, Jarrad M. Scarlett, Gregory J. Morton, Michael W. Schwartz

**Affiliations:** 1 Department of Medicine, University of Washington Medicine Diabetes Institute, Seattle, Washington, USA.; 2 Ivy Brain Tumor Center, Department of Neurosurgery, Barrow Neurological Institute, Phoenix, Arizona, USA.; 3 Department of Pediatric Gastroenterology and Hepatology, Seattle Children’s Hospital, Seattle, Washington, USA.

**Keywords:** Endocrinology, Metabolism, Diabetes

## Abstract

Growing evidence suggests that the pathogenesis of type 2 diabetes (T2D) involves dysfunctional central mechanisms, and, hence, the brain can be targeted to treat this disease. As an example, a single intracerebroventricular (icv) injection of fibroblast growth factor 1 (FGF1) can normalize hyperglycemia for weeks or months in rodent models of T2D. Convergent evidence implicates inhibition of a particular subset of neurons as a mediator of this FGF1 effect. Specifically, AgRP neurons, which are located in the hypothalamic arcuate nucleus (ARC) and are hyperactive in Lep*^ob/ob^* mice and other rodent models of T2D. To investigate whether chronic AgRP neuron inactivation mimics the antidiabetic action of FGF1, we directed an adeno-associated virus (AAV) containing a cre-inducible tetanus toxin–GFP (TeTx-GFP) cassette (or cre-inducible AAV GFP control) to the ARC of obese, diabetic male Lep*^ob/ob^* mice in which cre recombinase is expressed solely by AgRP neurons (Lep*^ob/ob^* AgRP-Cre mice). We report that over a 10-wk period of observation, hyperglycemia was fully normalized by AgRP neuron inactivation. In contrast, changes in energy homeostasis parameters (food intake, energy expenditure, body weight, and fat mass) were not observed. We conclude that in diabetic male Lep*^ob/ob^* mice, AgRP neuron hyperactivity is required for hyperglycemia but is dispensable for obesity.

## Abstract 2

Our finding that in diabetic Lep*^ob/ob^* mice, hyperglycemia is normalized by AgRP neuron inactivation identifies these neurons as key drivers of their diabetes.

## Introduction

Work in recent decades implicates the brain both in the pathogenesis of type 2 diabetes (T2D) and as a target for its treatment (1, 2). Although based largely on preclinical evidence, the relevance of these findings to human T2D is also growing (3). Distinct afferent/sensory, integrative/processing, and efferent/motor components of this central control system continue to be identified and add to evidence of a key role for the brain in both normal and abnormal glucose homeostasis (3). Numerous preclinical studies also demonstrate that the brain can be targeted to normalize the biologically defended level of glycemia across multiple diabetic animal models (3). Yet, the specific neuronal subsets responsible for these effects remain to be identified. To address this knowledge gap, the current work investigates the role played by a specific subset of neurons — AgRP neurons located in the hypothalamic arcuate nucleus (ARC) — in the pathogenesis of T2D.

The leptin-deficient Lep*^ob/ob^* mouse is a widely used model of T2D. In these mice, we reported in 2016 that a single intracerebroventricular (icv) injection of fibroblast growth factor 1 (FGF1) can induce diabetes remission lasting months (4). A series of subsequent studies identified AgRP neuron inhibition as a potential mediator of this sustained antidiabetic effect. For example, the antidiabetic effect of FGF1 was localized to the ARC and shown to require intact melanocortin 4 receptor (Mc4r) signaling (5), which is blocked by AgRP neuron activation (6). Since AgRP neurons are both hyperactive (6, 7) and inhibited in a prolonged manner following icv FGF1 injection in Lep*^ob/ob^* mice (8), we hypothesized a key role for durable inhibition of these neurons in the sustained antidiabetic effect elicited by icv FGF1 injection.

In support of this hypothesis, we report that permanent AgRP neuron inactivation recapitulates the sustained diabetes remission induced by icv FGF1 in Lep*^ob/ob^* mice. As this effect occurred despite having no detectable impact on food intake, energy expenditure, body weight, or body fat mass, our findings identify AgRP neurons as key drivers of hyperglycemia, but not obesity, in this T2D model.

## Results

### Targeting validation.

**Figure 1:**
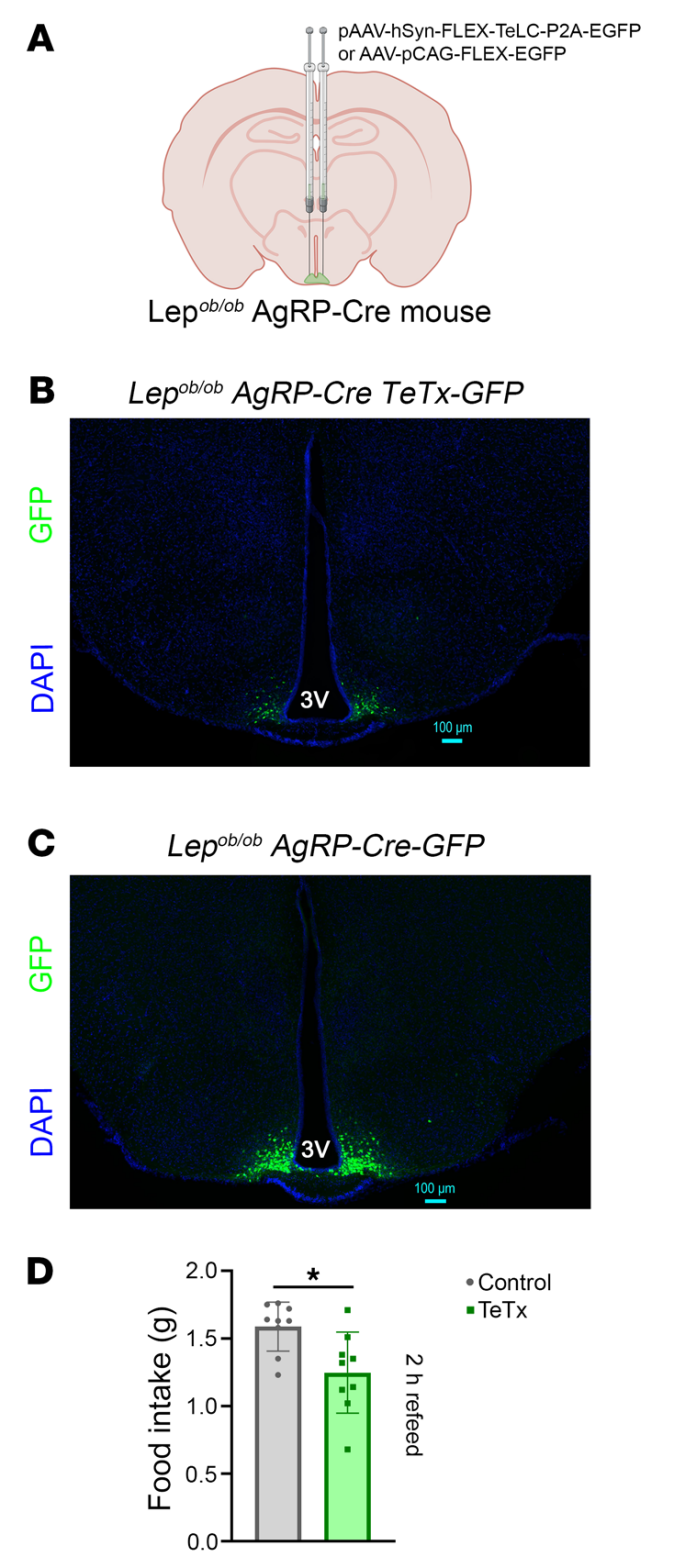
Validation of AgRP neuron inactivation. (**A**). Schematic depiction for chronic inactivation of AgRP neurons by microinjection of an AAV containing Cre-dependent GFP-fused TeTx delivered bilaterally to the arcuate nucleus (ARC) of Lep*^ob/ob^* AgRP-Cre mice relative to a fluorescent reporter control. Stereological fluorescent images from representative animals showing (**B**) GFP:TeTx and (**C**) GFP expression in Lep*^ob/ob^* AgRP-Cre mice. Scale bars: 100 μm. (**D**) GFP:TeTx Lep*^ob/ob^* AgRP-Cre mice (*n* = 9) exhibited a blunted refeeding response following an overnight fast when compared with those receiving the cre-inducible GFP controls (*n* = 9). 3V = third ventricle. Data are expressed as mean ± SEM, *P* versus GFP control as determined by 2-tailed t-test. *P < 0.05.

To permanently inactivate AgRP neurons, a cre-inducible tetanus toxin–GFP (TeTx-GFP) AAV vector was microinjected into the ARC of Lep*^ob/ob^* AgRP-Cre mice. Control mice received an injection of AAV containing only a cre-inducible GFP reporter (Figure 1A). Viral targeting accuracy was evaluated in both groups by detection of cre recombinase–dependent GFP fluorescence solely in the ARC (Figure 1, B and C). Three mice (out of a total of 21) were excluded on the basis of inaccurate bilateral targeting. Behavioral confirmation was provided by demonstrating a significant reduction of the refeeding response after an overnight fast among mice receiving the cre-inducible TeTx-GFP AAV compared with cre-inducible GFP controls (Figure 1D).

### Effect of AgRP neuron inactivation on food intake, body weight, blood glucose, and plasma insulin levels over 10 weeks in diabetic Lep^ob/ob^ mice.

**Figure 2:**
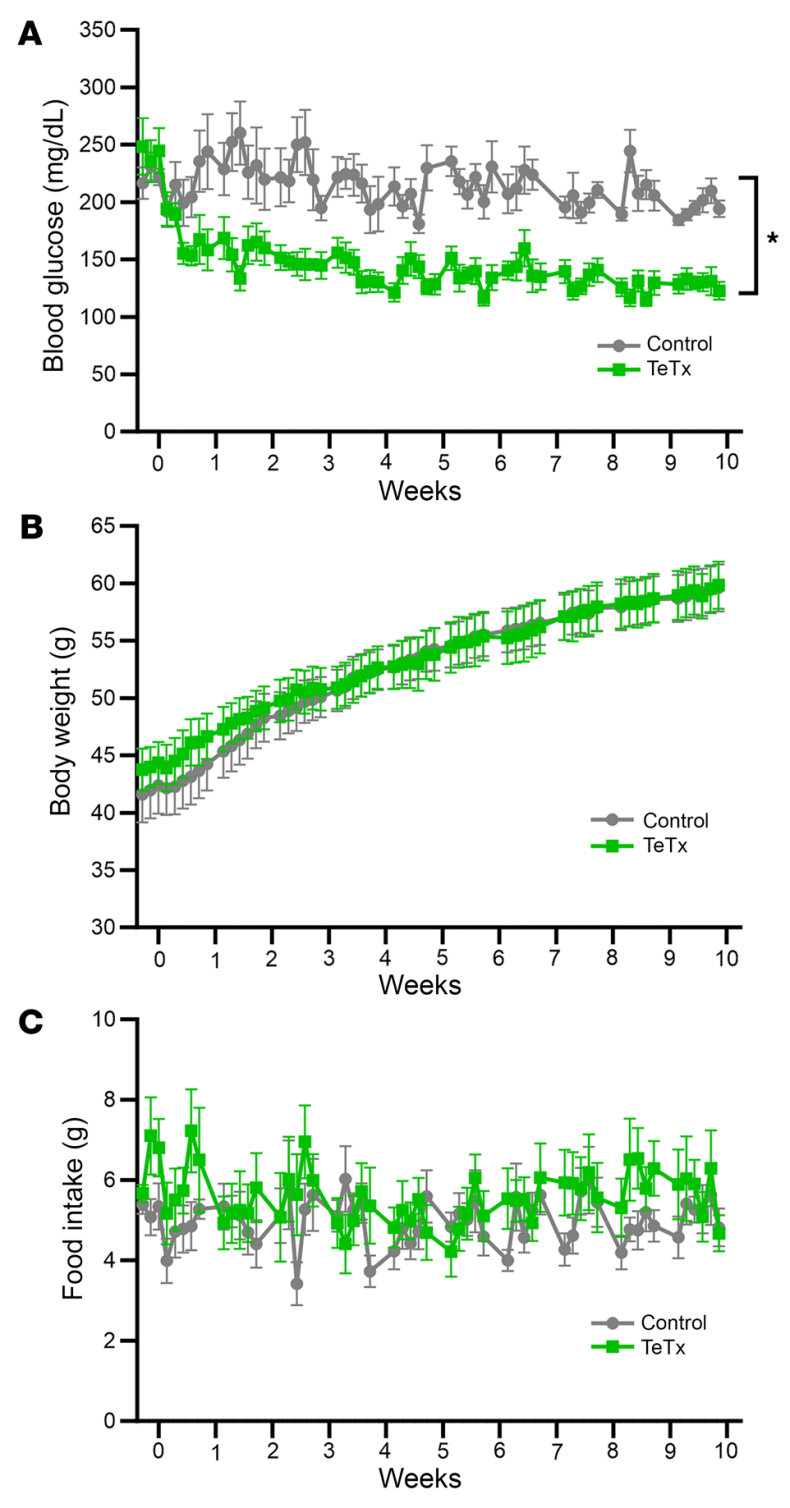
Permanent inactivation of AgRP neurons in Lep*^ob/ob^* AgRP-Cre mice induces diabetes remission independent of changes in body weight and food intake. (**A**) Nonfasted blood glucose, (**B**) body weight, and (**C**) food intake over 10 weeks in Lep*^ob/ob^* AgRP-Cre mice that received a bilateral injection to the arcuate nucleus (ARC) of a Cre-dependent GFP:TeTx (TeTx; *n* = 9) relative to a GFP control (Control; *n* = 9). Data are expressed as mean ± SEM, and *P* versus GFP control was determined by mixed model with the Geisser-Greenhouse correction. *P < 0.05.

The primary goal of this study was to determine the effect of AgRP neuron inactivation on serial measures of blood glucose over time in a mouse model of T2D. Among diabetic Lep*^ob/ob^* mice in which AgRP neurons were inactivated following microinjection of a cre-inducible TeTx-GFP AAV vector into the ARC, the mean blood glucose level fell from 230 mg/dL to 140 mg/dL within the first week and was stably maintained at this level over the next 9 weeks. In contrast, the mean daily blood glucose level of diabetic Lep*^ob/ob^* GFP controls remained well above 200 mg/dL for the entire 10-week period of observation (Figure 2A).

**Figure 3:**
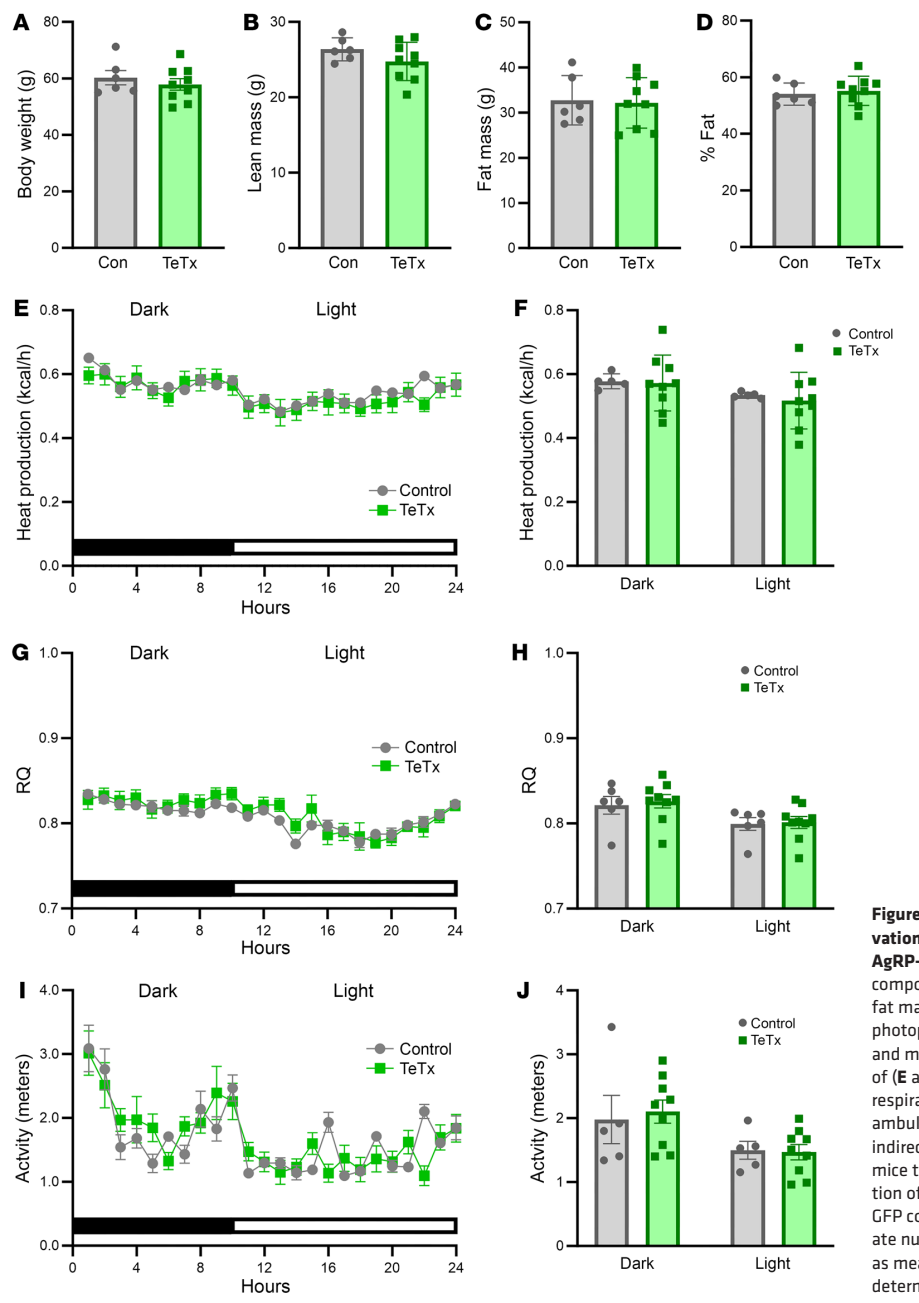
Effect of AgRP neuron inactivation on energy homeostasis in Lep*^ob/ob^* AgRP-Cre mice. (**A**) Body weight, body composition, including (**B**) lean mass, (**C**) fat mass, and (**D**) percentage of fat, and photoperiod-averaged 24-hour profiles and mean dark and light cycle measures of (**E** and **F**) heat production, (**G** and **H**) respiratory quotient (RQ), and (**I** and **J**) ambulatory activity as determined using indirect calorimetry in Lep*^ob/ob^* AgRP-Cre mice that received a bilateral microinjection of GFP:TeTx (TeTx; *n* = 9) relative to a GFP control (Control; *n* = 5–9) to the arcuate nucleus (ARC). Data are expressed as mean ± SEM, versus GFP control as determined by 2-tailed *t* test.

This outcome cannot be explained by reductions of either food intake or body weight, as neither parameter differed significantly between groups over the 10-wk course of the study (Figure 2, B and C). In addition, AgRP neuron inactivation had no effect on body composition (Figure 3, A–D), energy expenditure (Figure 3, E and F), respiratory quotient (Figure 3, G and H), or ambulatory activity (Figure 3, I and J). Thus, sustained normalization of glycemia induced by AgRP neuron inactivation in diabetic Lep*^ob/ob^* mice is mediated independently of changes in food intake, energy expenditure, body weight, or fat mass.

**Figure 4:**
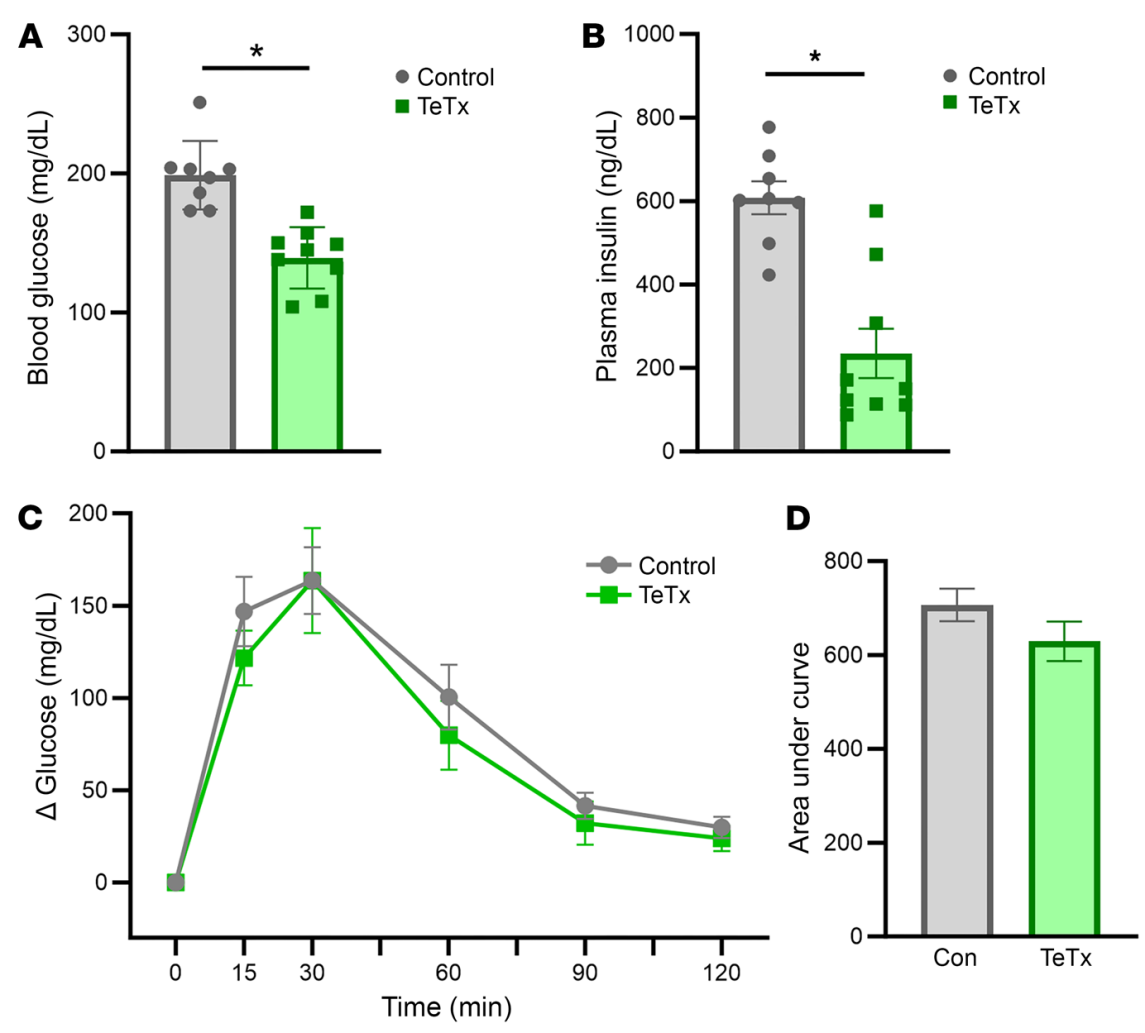
Effect of AgRP neuron inactivation on glucose tolerance in Lep*^ob/ob^* AgRP-Cre mice. (**A**) Blood glucose and (**B**) plasma insulin levels, (**C**) changes in blood glucose levels and (**D**) area under the glucose curve (AUC) during an intraperitoneal glucose tolerance test (ipgtt; 0.5g/kg BW) in Lep*^ob/ob^* AgRP-Cre mice following microinjection of GFP:TeTx (*n* = 9) or GFP control (*n* = 8–9) to the arcuate nucleus (ARC). Data are expressed as mean ± SEM, *P* versus GFP control as determined by 2-tailed *t* test. *P < 0.05.

Further analysis of the glucose metabolic phenotype elicited by AgRP neuron inactivation in diabetic Lep*^ob/ob^* mice revealed that the reduction in blood glucose levels was accompanied by a pronounced reduction in plasma insulin levels (Figure 4, A and B). Thus, increased insulin secretion is unlikely to explain the sustained remission of hyperglycemia. Similar to icv FGF1-treated mice and rats (4, 9), inactivation of AgRP neurons also had no effect on glucose tolerance, as determined by an intraperitoneal glucose tolerance test (ipGTT), as significant differences in the AUC_glucose_ curve were not detected between the 2 study groups (Figure 4, C and D).

### Potential mechanism(s) mediating the effect of AgRP neuron inactivation to normalize diabetic hyperglycemia in diabetic Lep^ob/ob^ mice.

**Figure 5:**
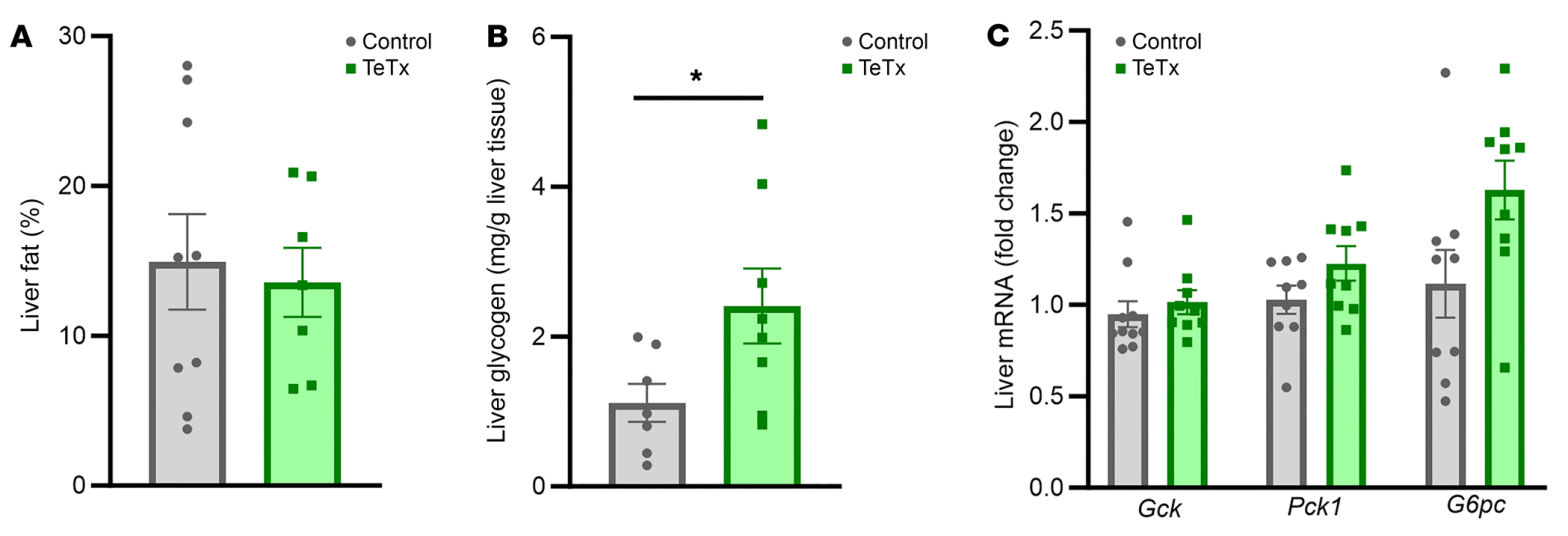
Effect of AgRP neuron inactivation on liver metabolism in Lep*^ob/ob^* AgRP-Cre mice. (**A**) Liver fat content, (**B**) liver glycogen content, and (**C**) hepatic mRNA levels of liver glucoregulatory genes glucokinase (*Gck*), phosphoenolpyruvate carboxykinase (*Pck1*), and glucose-6-phosphatase (*G6Pc*) using real-time PCR in Lep*^ob/ob^* AgRP-Cre mice that received microinjection of GFP:TeTx (*n* = 7–9) or GFP control (*n* = 7–9) to the arcuate nucleus (ARC). Data are expressed as mean ± SEM, *P* versus GFP control as determined by 2-tailed *t* test. *P < 0.05.

To investigate potential peripheral mechanisms involved in the effect of AgRP neuron inactivation to lower basal blood glucose and insulin levels, we examined liver fat and glycogen content as well as hepatic glucoregulatory gene expression (using real-time PCR). While reduced liver fat content is often associated with improvements in insulin sensitivity and glycemic control (10), no significant differences were observed between Lep*^ob/ob^* TeTx-GFP–treated mice and their GFP controls (Figure 5A). Similarly, neither hepatic expression of the key glucoregulatory enzyme glucokinase (*Gck*) or the gluconeogenic genes phosphoenolpyruvate carboxykinase (*Pck1*) and glucose-6-phosphatase (*G6Pase*) differed between groups (Figure 5C). In contrast, liver glycogen content was significantly increased (Figure 5B), as has been observed following icv FGF1 injection in Lep*^ob/ob^* mice (4).

**Figure 6:**
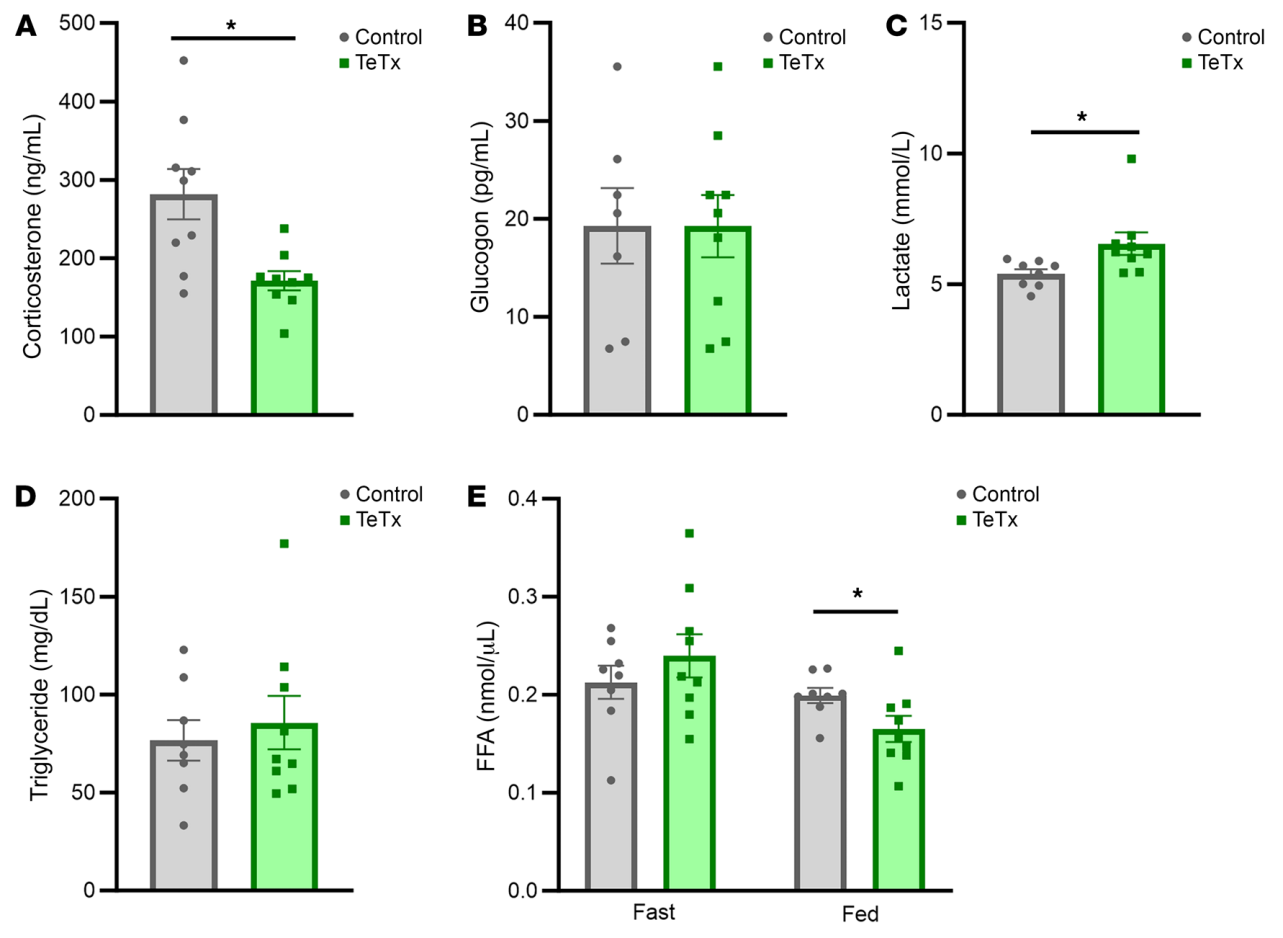
Inactivation of AgRP neurons lowers corticosterone levels in Lep*^ob/ob^* AgRP-Cre mice. Plasma levels of (**A**) corticosterone and (**B**) glucagon during the mid-light cycle in the fed state. Plasma (**C**) lactate, (**D**) triglyceride (TG), and (**E**) free fatty acid levels in either the fed or 6-hr fasted state in Lep*^ob/ob^* AgRP-Cre mice that received microinjection of GFP:TeTx (*n* = 9) or GFP control (*n* = 7–9) to the arcuate nucleus (ARC). Data are expressed as mean ± SEM, *P* versus GFP control as determined by 2-tailed *t* test. *P < 0.05.

Elevated levels of plasma corticosterone and glucagon levels have each been reported in diabetic Lep*^ob/ob^* mice (11–14) and both are implicated in diabetes pathogenesis (15, 16). We found that in Lep*^ob/ob^* mice, plasma corticosterone levels were significantly reduced in mice with AgRP neuron inactivation relative to controls, whereas plasma glucagon levels were not significantly affected (Figure 6, A and B). Thus, suppression of hypercorticosteronemia (but not hyperglucagonemia) may contribute to the glucose-lowering effect elicited by inactivation of AgRP neurons. We also report that, as observed in icv FGF1-treated Lep*^ob/ob^* mice (4, 9), plasma lactate levels were elevated in ARC^AgRP^-inactivated mice relative to GFP control mice (Figure 6C), an effect that has been linked to increased hepatic glucose uptake (4, 9). Conversely, plasma free fatty acid (FFA) levels were reduced in the fed, but not the fasted, state in TeTx:GFP-treated Lep*^ob/ob^* mice versus controls, whereas group differences in plasma triglyceride levels were not observed (Figure 6, D and E).

## Discussion

The current work was undertaken to investigate neural mechanisms underlying diabetes pathogenesis: namely, the contribution made by excessive activity of hypothalamic AgRP neurons. We report that, in diabetic Lep*^ob/ob^* mice, a widely used model of T2D, selective TeTx-mediated inactivation of AgRP neurons was sufficient to normalize diabetic hyperglycemia for at least 10 weeks. Since these neurons are hyperactive in Lep*^ob/ob^* mice (6, 7) and other rodent diabetes models (17–19), we conclude that excessive AgRP neuron activity is a key driver of the diabetes phenotype of these animals. In contrast, AgRP neuron inactivation had no detectable impact on any energy homeostasis parameter (food intake, energy expenditure, respiratory quotient, body weight, or body fat mass). We conclude from these findings that AgRP neuron hyperactivity is required for the hyperglycemia of diabetic Lep*^ob/ob^* mice but is dispensable for their hyperphagia and obesity.

Our focus on the role of the brain as a target for the treatment of T2D originated with the unexpectedly durable antidiabetic action of centrally administered FGF1. First reported in 2016 (4), a single icv FGF1 injection was shown to normalize glycemia for weeks or months across multiple rodent models of T2D (including Lep*^ob/ob^* mice). Subsequent studies identified the ARC, which is where AgRP neurons are located, as the brain area responsible for this FGF1 effect (20) and established a key role for the melanocortin system in this response. Specifically, the antidiabetic action of FGF1 is blocked by either pharmacological or genetic disruption of Mc4r signaling (5). As AgRP neuron activation also blocks Mc4r signaling, and as these neurons are hyperactive in Lep*^ob/ob^* mice (6, 7), we identified them as potential targets for the antidiabetic action of FGF1. Consistent with this hypothesis, AgRP neurons are inhibited for at least 2 weeks following a single icv FGF1 injection (8).

In the current work we report not only that hyperglycemia is normalized comparably by icv FGF1 and AgRP neuron inactivation, but that in each case the effects are sustained for weeks or months. In addition, these effects are mediated independently of long-term changes in food intake or body fat mass; changes in serum triglyceride or plasma glucagon levels; or improvements in either glucose tolerance or hepatic steatosis. Similar to icv FGF1 injection (4, 9), inactivation of AgRP neurons is also associated with increases of both liver glycogen content and plasma lactate levels, findings suggestive of increased hepatic glucose uptake and intrahepatic glycolysis (4, 9).

Despite these similarities, some aspects of the response to AgRP neuron inactivation were not observed following icv FGF1 injection. Notable among these was the finding of reduced plasma corticosterone levels. Given that HPA axis hyperactivity is a well-documented consequence of congenital leptin deficiency in these mice (12–14), we infer from this observation that excessive AgRP neuron activity, which is also a consequence of leptin deficiency, is required for this pathological response. This interpretation is strengthened by evidence that during fasting, HPA axis activation is causally linked to AgRP neuron activation. Specifically, GABAergic neurons in the bed nucleus of the stria terminalis (BNST) that project onto and inhibit corticotropin-releasing hormone (CRH) neurons are themselves inhibited by projections from AgRP neurons lying upstream (21). Consequently, AgRP neuron activation disinhibits CRH neurons, thereby stimulating glucocorticoid secretion.

Since glucocorticoid excess (Cushing’s syndrome) predisposes one to obesity and diabetes both in humans and in animal models (22), these findings support the hypothesis that reduced HPA axis activity may have contributed to sustained glucose lowering induced by AgRP neuron inactivation. This interpretation, however, raises a key question: how would a decrease of circulating glucocorticoid levels ameliorate hyperglycemia, but not obesity? While the answer is unknown, we note that, although plasma corticosterone levels were lowered by AgRP neuron inactivation, they remained higher than is typical for a WT mouse. It is therefore possible that corticosterone levels were lowered sufficiently to ameliorate hyperglycemia, but not to ameliorate hyperphagia or obesity.

Also somewhat paradoxical is the finding that, despite its clear inhibitory effect on AgRP neuron activity (8, 23), icv FGF1 injection does not lower corticosterone levels in Lep*^ob/ob^* mice (9). Relevant to this finding is that some AgRP neurons are not inhibited by FGF1 (8, 23). It is therefore conceivable that while the subset of AgRP neurons that drive HPA axis activity is targeted by our TeTx-based inactivation strategy, these neurons are not among those inhibited by FGF1. These considerations highlight important unanswered questions about interactions between FGF1, AgRP neurons, the HPA axis, and the pathogenesis of obesity and diabetes.

We also report that in Lep*^ob/ob^* mice, normalization of hyperglycemia by AgRP neuron inactivation was associated with markedly decreased plasma insulin levels. Implicit in this observation is that insulin secretion declines in response to AgRP neuron inactivation in these animals — a finding in sharp contrast to the absence of any detectable change of food intake or obesity. Thus, we infer that the pronounced hyperinsulinemia characteristic of these animals (24, 25) depends in part on AgRP neuron hyperactivity, and that this effect is not secondary to hyperphagia or obesity. Interestingly, the inhibitory effect of leptin on AgRP neurons in Lep*^ob/ob^* mice associates with similar reductions of circulating glucose and insulin levels — although food intake and body weight are also reduced by leptin administration (25). Table 1 summarizes the effects of leptin, FGF1, and AgRP neuron inactivation on parameters relevant to energy and glucose homeostasis.

Located adjacent to one another in the ARC, AgRP and POMC neurons have opposing effects on Mc4r signaling in neurons lying downstream (26). Thus, whereas POMC neurons release the Mc4r agonist α-melanocyte stimulating hormone (αMSH), AgRP is an inverse agonist that inhibits Mc4r signaling (6). In addition to these opposing effects, these 2 neuronal subsets are also regulated in a reciprocal manner by diverse inputs, among them leptin and FGF1. Thus, both leptin (27–32) and FGF1 (8, 23) inhibit AgRP while stimulating POMC neurons, thereby increasing net Mc4r signaling.

Conversely, leptin-deficient states (including fasting as well as most forms of diabetes) are characterized by hyperactive AgRP neurons while POMC neurons are inhibited. By potently inhibiting Mc4r signaling, this combination is implicated in the hyperphagia, obesity, and diabetes of leptin-deficient models, and reversal of this effect is implicated in the restoration of euglycemia to diabetic animals following central administration of either leptin (33) or FGF1 (5). Interestingly, icv leptin administration also normalizes excessive glucagon secretion and HPA axis activity in uncontrolled diabetes (34), whereas icv FGF1 injection does not — and yet, both appear to inhibit AgRP neurons, activate POMC neurons, and thereby increase net melanocortin signaling.

With this background, it seems somewhat paradoxical that, unlike central leptin administration, neither icv FGF1 injection nor chronic AgRP neuron inactivation detectably impact long-term energy balance (food intake, energy expenditure, body weight, or fat mass), despite eliciting sustained normalization of hyperglycemia. Here, we consider 3 related questions raised by this paradox: (a) does AgRP neuron inhibition mediate leptin-induced anorexia; (b) does leptin act directly on AgRP neurons to inhibit them, or indirectly by activating inhibitory neurons lying upstream; and (c) are hyperphagia and obesity in leptin-deficient mice driven by activation of AgRP neurons, non-AgRP neurons, or both?

Published studies do not offer definitive answers to these questions. On the one hand, hyperphagia, obesity, and hyperglycemia were reported following acute, CRISPR-mediated leptin receptor deletion specifically from AgRP neurons in adult mice (19). This finding suggests that both energy and glucose homeostasis are constrained by direct, leptin-mediated inhibition of these neurons. A similar outcome is reported following germline deletion of leptin receptors from broad subsets of hypothalamic neurons (that include AgRP neurons, e.g., GABAergic neurons, or neurons that express the transcription factor Nkx2.1 during development (35)). Importantly, however, these effects are not recapitulated by germline deletion of leptin receptors solely from AgRP neurons (36). Together, these findings suggest that, while AgRP neuron activity in adult mice is constrained by the direct, inhibitory of action of leptin, these neurons are also inhibited via actions of leptin on other neurons (e.g., activation of GABAergic neurons lying upstream (37)).

Our data extend these findings. Implied in our finding that in Lep*^ob/ob^* mice, AgRP neuron inactivation has no detectable effect on any energy homeostasis parameter, is that leptin’s potent effects on food intake and body weight in these mice must involve other neurons. Thus, we hypothesize that in these mice, hyperphagia and obesity — but not hyperglycemia — are driven by non-AgRP neurons. While the identity of these neurons is still awaited, our findings suggest that they (a) are activated by leptin deficiency and thus are inhibited by leptin administration; (b) are sufficient to drive hyperphagia and obesity in the absence of functional AgRP neurons; and (c) are not targets for the action of FGF1. This interpretation is consistent with recent evidence that in otherwise normal mice, selective ablation of AgRP neurons has little effect on overall energy homeostasis (although it does blunt the refeeding response to a fast (38), as we observed following AgRP neuron inactivation). In addition, activation of non-AgRP, GABAergic neurons in the ARC reportedly drives severe obesity in mice (39). Identifying non-AgRP neuronal subsets that are responsible for hyperphagia and obesity — but are insufficient to drive hyperglycemia — in leptin-deficient states is a priority for future research.

Discussion of the role played by AgRP neurons in diabetes pathogenesis would be incomplete without acknowledging the 2 additional signaling molecules — neuropeptide Y (NPY) and GABA — that are expressed by and synaptically released from these neurons. Hyperactivity of AgRP neurons therefore increases signaling by NPY and GABA as well as by AgRP. Beyond being a potent orexigen (40), NPY action in the brain is reported to cause insulin resistance associated with hyperinsulinemia and reduced glucose uptake by skeletal muscle (41, 42). In addition, NPY appears to contribute to insulin resistance induced by acute stimulation of AgRP neurons, and GABA release from AgRP neurons has been shown to inhibit stress-activated, anorexia-inducing neurons located in the parabrachial nucleus (43). The unique contribution(s) to normal and abnormal glucose homeostasis made by synaptic release of AgRP, NPY, and GABA from AgRP neurons awaits further study.

To the extent that the effects of AgRP neuron inactivation reported herein translate to human diabetes, our finding that this intervention ameliorates hyperglycemia without apparent off-target effects identifies these neurons as potential therapeutic targets. In this context, we note that, in normal mice, AgRP neurons are rapidly inhibited following systemic administration of agonists of either GLP1 or GIP receptors (44). Given the impressive antidiabetic efficacy of this class of drugs, it will be of interest in future studies to determine whether AgRP neuron inhibition contributes to their therapeutic benefit. If so, selective, durable AgRP neuron inhibition may prove an attractive strategy for the future treatment of T2D in humans.

## Methods

### Sex as a biological variable

Our study exclusively examined male mice. Additional studies are required to determine whether our findings are relevant for female mice.

### Animals

To permanently inactivate AgRP neurons in a mouse model of T2D, we crossed AgRP-IRES-Cre (AgRP*^tm1(cre)Lol/J^*) mice (Jackson Laboratory, Strain no.: 012899) which were originally generated, as reported previously (45), onto Lep*^ob/+^* (B6.Cg-*Lep^ob^/*J) mice (Jackson Laboratory, Strain no.: 000632), to generate Lep*^ob/ob^* AgRP-Cre mice. Only adult male, littermate mice were studied. Prior to study, all animals were individually housed in a temperature-controlled room on a 14:10 light:dark cycle under specific pathogen-free conditions with ad libitum access to drinking water and standard laboratory chow (LabDiet), unless otherwise stated.

#### Stereotaxic surgeries and viral injections.

AgRP neurons were permanently rendered functionally inactive in diabetic Lep*^ob/ob^* AgRP-Cre mice by bilateral microinjection into the ARC of an AAV containing a cre-inducible TeTx-GFP cassette (*n* = 10): pAAV-hSyn-FLEX-TeLC-P2A-EYFP-WPRE (Addgene, Plasmid no. 135391 (46, 47)). Control littermate Lep*^ob/ob^* AgRP-Cre mice received a cre-inducible AAV containing GFP only (*n* = 11) (pCAG-FLEX-EGFP-WPRE; Addgene, Plasmid no. 51502). The ARC was targeted using the following coordinates: AP: −1.4 mm; ML: ±0.3 mm; DV: −5.5 mm, and AAV was microinjected at a rate of 60 nL/min for 5 minutes (300 nL total volume), followed by a 5 minute pause and slow withdrawal. All animals received a perioperative subcutaneous injection of buprenorphine hydrochloride (0.05 mg/mL; Reckitt Benckiser). Of an initial cohort of 21 Lep*^ob/ob^* AgRP-Cre mice, 2 mice from the AAV GFP control group and 1 from the TeTx group were excluded based on inaccurate targeting. This resulted in a sample size of 9 in each of the 2 groups, and a targeting efficiency of 86%. Impaired AgRP neuron function was documented by reduced food intake over a 2 hour period following an overnight fast.

#### Criteria for sustained diabetes remission.

The effect of TeTx-mediated AgRP neuron inactivation on the blood glucose level of Lep*^ob/ob^* AgRP-Cre was compared with GFP controls over a 10 week period of observation. Consistent with previously publications (4, 9), sustained remission of diabetes was defined as continuous maintenance of blood glucose levels under 200 mg/dL. Glucose levels were measured using a hand-held glucometer (ACCU-CHEK, Roche Diabetes Care Inc.) on tail capillary blood obtained 2 hours after light cycle onset under nonfasted conditions. Daily food intake, body weight, and blood glucose levels were monitored over a period of 10 weeks.

#### Intraperitoneal glucose tolerance testing.

Intraperitoneal glucose tolerance tests (ipGTTs) were conducted in 6 hour–fasted animals by measuring blood glucose levels at t = 0, 15, 30, 60, 90, and 120 minutes from a tail capillary blood sample using a hand-held glucometer (ACCU-CHEK, Roche Diabetes Care Inc.) following an i.p. injection of glucose (15% dextrose) at a dose of 0.5 g/kg body weight.

#### Plasma, body, and tissue composition analysis.

Blood samples were collected into EDTA-treated tubes, centrifuged, and plasma removed for measurement of immunoreactive insulin, glucagon, and corticosterone by ELISA (Crystal Chem Inc.). Levels of plasma lactate (Abcam), free fatty acids (Abcam), triglycerides (RayBiotech) and liver glycogen (Abcam) were determined by enzymatic colorimetric assays. Liver glycogen content was normalized to grams wet weight after the colorimetric assay, and liver fat content was expressed as a percent of total liver mass. Total body composition and liver fat mass was measured using quantitative magnetic resonance spectroscopy (EchoMRI 3-in-1; Echo MRI) with support from the NIDDK-funded Nutrition Obesity Research Center (NORC) Energy Balance Core at the University of Washington (48).

#### Indirect calorimetry.

For indirect calorimetry studies, Lep*^ob/ob^* AgRP-Cre mice were acclimated to metabolic cages after which energy expenditure was measured using a computer-controlled indirect calorimetry system (Promethion; Sable Systems) with support from the NIH-funded NORC Energy Balance Core, as previously described (46, 47, 49). For each animal, O_2_ consumption and CO_2_ production were measured at 5 minute intervals and respiratory quotient was calculated as the ratio of CO_2_ production to O_2_ consumption. Energy expenditure was calculated using the Weir equation based on the rate of O_2_ consumption (VO_2_) (50). Ambulatory activity was measured continuously, with consecutive adjacent infrared beam breaks in the x-, y-, and z- axes scored as an activity count that was recorded every 5 minutes. Data acquisition and instrument control were coordinated by MetaScreen v.1.6.2 and raw data was processed using ExpeData v.1.4.3 (Sable Systems) using an analysis script documenting all aspects of data transformation.

#### IHC detection of GFP.

Animals were anesthetized with ketamine:xylazine and perfused with 1× PBS followed by 4% (v/v) paraformaldehyde in 0.1M PBS. Brains were removed and postfixed for 24 hours in paraformaldehyde, followed by sucrose (30%) dehydration and embedded in OCT compound blocks. Anatomically matched free-floating coronal sections (35 μm thickness) from the rostral-to-caudal extent of the hypothalamus were collected and stored in 1× PBS with 0.02% sodium azide at 4°C. Free-floating sections were washed in PBS with 0.1% Triton (PBS-T) at room temperature 5 times for 10 minutes each time and then incubated overnight at 4°C with chicken anti-GFP antibody (1:5,000; GFP1020; Aves Labs), followed by incubation in donkey anti-chicken Alexa Fluor 488 (1:1,000; 703-545-155; Jackson ImmunoResearch). To provide a nuclear contrast stain, sections were incubated with DAPI (1:1000; MBD0015; Sigma–Aldrich) in PBS for 10 minutes at room temperature, followed by 3 washes in PBS-T and 2 additional washes in PBS. Sections were then mounted with polyvinyl acetate.

#### RT–PCR.

Individual liver tissue samples were homogenized and RNA was isolated using Qiagen RNeasy Micro Kit (Kit 57805496, Hilden) and isolated RNA concentrations were quantified by Nanodrop (Thermo Fisher Scientific). Levels of specific transcripts were quantified by real-time PCR (ABI Prism 7900 HT; Applied Biosystems) using SYBR Green (Applied Biosystems) and the following specific primers: *Gck* (forward*-CAAGCTGCACCCGAGCTT;* reverse*-TGATTCGATGAAGGTGATTTCG*), *Pck1* (forward*-GGCGGAGCATATGCTGATCC*; reverse*-CCACAGGCACTAGGGAAGGC*), *G6pc* (forward*-TCAACCTCGTCTTCAAGTGGATT*; reverse*-CTGCTTTATTATAGGCACGGAGCT*). Expression levels of each gene were normalized to a house keeping gene (18S RNA) and standard curve. Nontemplate controls were incorporated into each PCR run.

### Statistics

Results are expressed as Mean ± SEM. Significance was established at *P* < 0.05, 2-tailed. Serial measures of blood glucose, body weight, food intake, and ipGTT data between the 2 study groups were compared using a group-by-time mixed factorial repeated measures ANOVA with the Geisser-Greenhouse correction (GraphPad Software). For intergroup comparisons, a 2-sample unpaired, 2-tailed Student’s *t* test was applied. The collected data satisfied the normality assumptions necessary for the statistical analyses performed.

### Study approval

All procedures were performed in accordance with the National Institutes of Health Guide for the Care and Use of Laboratory Animals and were approved by the Institutional Animal Care and Use Committee at the University of Washington, Seattle, WA.

### Data availability

Values for all data points in graphs are reported in the Supporting Data Values file (https://doi.org/10.1172/JCI189842DS1). This file includes all raw data for each figure presented in the study. Additional datasets generated during and/or analyzed in the current study are not publicly available but are available from the corresponding author upon request.

## Author contributions

YG, MG, JMS, GJM, and MWS designed the experiments. YG, MG, VD, CLF, CLB, BAP, AT, MKH, and JMS collected the data. YG, MG, VD, CLB, JMS, and GJM analyzed the data. YG, JMS, GJM, and MWS wrote the manuscript. All authors reviewed and edited the manuscript. MWS is the guarantor of this work and, thus, had full access to all the data in the study and takes responsibility for the accuracy and integrity of the data.

## Supplementary Material

Supporting data values

## Figures and Tables

**Table 1:**
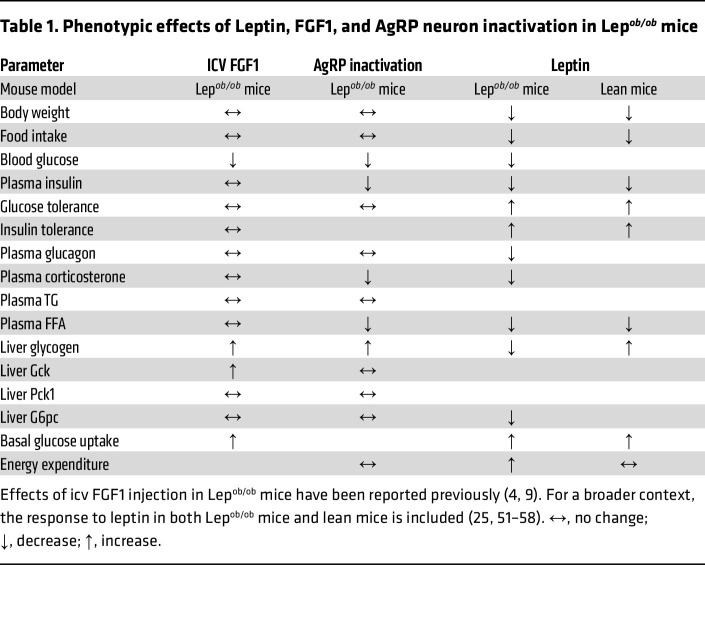
Phenotypic effects of Leptin, FGF1, and AgRP neuron inactivation in Lep*^ob/ob^* mice

## Version 1 05/15/2025

Electronic publication
